# Secreted Frizzled-Related Protein 4 (SFRP4) Is an Independent Prognostic Marker in Prostate Cancers Lacking *TMPRSS2: ERG* Fusions

**DOI:** 10.1007/s12253-020-00861-9

**Published:** 2020-07-16

**Authors:** Christian Bernreuther, Ferdous Daghigh, Katharina Möller, Claudia Hube-Magg, Maximilian Lennartz, Florian Lutz, Sebastian Dwertmann Rico, Christoph Fraune, David Dum, Andreas M. Luebke, Till Eichenauer, Christina Möller-Koop, Thorsten Schlomm, Corinna Wittmer, Hartwig Huland, Hans Heinzer, Markus Graefen, Alexander Haese, Eike Burandt, Maria Christina Tsourlakis, Till S. Clauditz, Doris Höflmayer, Jakob R. Izbicki, Ronald Simon, Guido Sauter, Sarah Minner, Stefan Steurer, Jan Meiners

**Affiliations:** 1 Institute of Pathology, University Medical Center Hamburg-Eppendorf, Martinistr, 52, 20246 Hamburg, Germany; 2 Department of Urology, University Medical Center Hamburg-Eppendorf, Hamburg, Germany; 3 Department of Urology, Charité-Universitätsmedizin Berlin, Berlin, Germany; 4 Martini-Clinic, Prostate Cancer Center, University Medical Center Hamburg-Eppendorf, Hamburg, Germany; 5 General, Visceral and Thoracic Surgery Department and Clinic, University Medical Center Hamburg-Eppendorf, Hamburg, Germany

**Keywords:** SFRP4, Prognosis, Prostate cancer, TMA

## Abstract

Secreted frizzled-related protein 4 (SFRP4) controls WNT signaling and is thought to play a role for tumor aggressiveness. Here, we analyzed a tissue microarray containing 11,152 prostate cancers with pathological, clinical and molecular data by immunohistochemistry. SFRP4 expression was higher in cancer than in non-neoplastic acinar cells. SFRP4 staining was seen in 64.9% of tumors and classified as weak in 33.2%, moderate in 23.9% and strong in 7.8% of cancers. SFRP4 overexpression was linked to advanced tumor stage, high classical/quantitative Gleason grade (*p* < 0.0001 each), lymph node metastasis (*p* = 0.0002), and a positive surgical margin (*p* = 0.0017). SFRP4 positivity was markedly more frequent in ERG positive (77.4%) than in ERG negative cancers (57.4% *p* < 0.0001). Subset analyses in 2725 cancers with and 3592 cancers without *TMPRSS2:ERG* fusion revealed that associations with tumor phenotype and patient outcome were largely driven by the subset of ERG negative tumors. In a multivariate analysis including various postoperative and prognostic clinico-pathological features, SFRP4 protein expression emerged as an independent prognostic parameter in ERG negative cancers. SFRP4 immunostaining was significantly linked with 10 of 11 previously analyzed chromosomal deletions (*p* < 0.05 each). In conclusion, high SFRP4 immunostaining is associated with poor prognosis and genomic instability in ERG negative prostate cancers.

**Electronic supplementary material:**

The online version of this article (10.1007/s12253-020-00861-9) contains supplementary material, which is available to authorized users.

## Introduction

In 2018 prostate cancer was the most common cancer in males in the majority of the countries of the world and the third most common cause of cancer related death [1]. While variable in the clinical course, a minority of patients needs aggressive therapy. Presently available criteria (Gleason grade, clinical stage and PSA value) are statistically powerful but do not permit clear-cut treatment decisions for every patient. It is hoped that molecular prognostic biomarkers more reliably predict disease outcome.

Secreted frizzled-related protein 4 (SFRP4) belongs to a family of 5 glycoproteins with a cysteine-rich domain which is homolog to the Wnt-binding domain of frizzled receptors. SFRPs function as extracellular inhibitors of Wnt signaling by sequestering Wnt ligands in the extracellular space [2, 3]. SFRP4 is physiologically expressed in the uterus, fallopian tubes and testis according to the Human Protein Atlas project [4], but aberrant expression and/or promoter methylation has been reported from many human cancer types including malignant mesotheliomas [5], ovarian- [6, 7], colon [8, 9], endometrial- [10], cervical- [11], bladder [12], pancreatic- [13] and other cancers (reviewed in [14, 15]). SFRP4 also appears to play a role in prostate cancer, although discrepant findings have been reported as to whether its loss or up-regulation associates with disease progression. Early studies found that SFRP4 overexpression was associated with a decreased rate of proliferation, decreased anchorage-independent growth, and decreased invasiveness in PC-3 and LNCaP cancer cells [16, 17], and that membranous SFRP4 expression was associated with good prognosis in 229 clinical prostate cancer specimens [16]. In contrast, other authors reported that cytoplasmic overexpression of SFRP4 was linked to poor prognosis in cohorts of 33–536 prostate cancers [18, 19]. SFRP4 up-regulation was also been found on the mRNA level in several studies [20–24] and SFRP4 is part of a commercial prostate cancer gene expression assay to estimate tumor aggressiveness [25].

Although previous findings are controversial, they raise the possibility that the SFRP4 protein may represent a useful prognostic biomarker for prostate cancer. To further investigate its prognostic role, a prostate cancer tissue microarray containing tumor samples from more than 11,000 individual patients was analyzed in this study.

## Materials and Methods

### Patients

Radical prostatectomy specimens were available from 11,152 patients, undergoing surgery between 1992 and 2012 at the Department of Urology and the Martini Clinics at the University Medical Center Hamburg-Eppendorf. Follow-up data were available for a total of 11,419 patients with a median follow-up of 49 months (range: 1 to 276 months; Table 1). Prostate specific antigen (PSA) values were regularly measured following surgery and PSA recurrence was defined as a postoperative PSA of 0.2 ng/ml and a subsequent increase. All prostate specimens were analyzed according to a standard procedure, including a complete embedding of the entire prostate for histological analysis [26]. Histopathological data were retrieved from the patients’ records, including pT, Gleason grade, pN and status of the resection margin. Quantitative Gleason grading [27] was performed by subdividing Gleason 3 + 4 and 4 + 3 cancers according to their percentage of Gleason 4. For practical use, we subdivided the 3 + 4 and 4 + 3 cancers in 8 subgroups: 3 + 4 ≤ 5% Gleason 4, 3 + 4 6–10%, 3 + 4 11–20%, 3 + 4 21–30%, 3 + 4 31–49%, 4 + 3 50–60%, 4 + 3 61–80% and 4 + 3 > 80% Gleason 4. In addition, separate groups were defined by the presence of a tertiary Gleason 5 pattern, including 3 + 4 Tert.5 and 4 + 3 Tert.5. The TMA manufacturing process was as described [28]. In short, one 0.6 mm core was taken from a tumor containing tissue block from each patient. The tissues were distributed among 27 TMA blocks, each containing 144 to 522 tumor samples. For internal controls, each TMA block also contained various control tissues, including normal prostate tissue. The attached molecular database included data on Ki67 labeling Index (Ki67LI) from 5492 tumors, expanded from [29], ERG protein expression from 8134 and ERG rearrangement analysis by fluorescence in situ hybridization (FISH) from 5515 tumors [30, 31], as well as deletion status of 3p13 *(FOXP1)* from 5503 tumors [32], 5q21 *(CHD1) f*rom 6145 tumors [33], 6q15 *(MAP3K7)* from 4663 tumors [34], 8p21 from 5556 tumors [35], 10q23 *(PTEN)* from 5158 tumors [36], 12p13 *(CDKN1B)* from 4887 tumors [37], 12q24 from 5721 tumors [38], 13q14 *(FOXO1, RB1)* from 5915 tumors [39], 16q24 from 4413 tumors [40], 17p13 *(TP53)* from 6437 tumors [41] and 18q21 from 5578 tumors [42].

**Table 1:** Pathological and clinical data of the arrayed prostate cancers

	Study Cohort on TMA	Biochemical relapse among categories
	(*n* = 11,152)	(*n* = 1824; 18.5%)
Follow-up (mo)		
Mean	59.4	–
Median	49.5	–
Age (y)		
≥50	323	51 (15.8%)
51–59	2696	445 (16.5%)
60–69	6528	1078 (16.5%)
≥70	1498	241 (16.1%)
Pretreatment PSA (ng/ml)		
<4	1417	142 (10.0%)
4–10	6866	823 (12.0%)
10–20	2160	525 (24.3%)
>20	719	308 (42.8%)
pT category (AJCC 2002)		
pT2	7514	565 (7.5%)
pT3a	2403	586 (24.4%)
pT3b	1265	623 (49.2%)
pT4	63	49 (77.8%)
Gleason grade		
≤3 + 3	2734	342 (12.5%)
3 + 4	5622	1057 (18.8%)
3 + 4 Tert. 5	379	84 (22.2%)
4 + 3	912	405 (44.5%)
4 + 3 Tert. 5	520	230 (44.2%)
≥4 + 4	416	221 (53.1%)
pN category		
pN0	6115	1126 (18.4%)
pN+	568	298 (52.5%)
Surgical margin		
Negative	8999	1148 (12.8%)
Positive	2096	639 (30.5%)

Numbers do not always add up to 11,152 in the different categories because of cases with missing data. *Abbreviation*: *AJCC* American Joint Committee on Cancer

Percentage (%) in the column “Biochemical relapse among categories” refers to the fraction of samples with biochemical relapse in the different parameter in the different categories

### Immunohistochemistry

Freshly cut TMA sections were immunostained on one day and in one experiment. Slides were deparaffinized and exposed to heat-induced antigen retrieval for 5 min in an autoclave at 121 °C in pH 7.8 Tris-EDTA-Citrate buffer. Primary antibody specific for SFRP4 (rabbit monoclonal antibody, clone [EPR9389], Abcam, Cambridge, UK; cat#154167; dilution 1:900) was applied at 37 °C for 60 min. Bound antibody was then visualized using the EnVision Kit (Dako, Glostrup, Denmark) according to the manufacturer’s directions. SFRP4 staining typically showed a uniformly intense granular cytoplasmic pattern in 100% of tumor cells of a tissue spot. Therefore, only the staining intensity of the tumor cells was evaluated according the following criteria: a) lack of staining was considered “negative”, b) 1+ intensity was considered “weak”, c) 2+ intensity was considered “moderate” and d) 3+ intensity was considered “strong”.

### Statistics

Statistical calculations were performed with JMP 12® software (SAS Institute Inc., NC, USA). Contingency tables and the chi^2^-test were performed to search for associations between molecular parameters and tumor phenotype. Survival curves were calculated according to Kaplan-Meier. The Log-Rank test was applied to detect significant differences between groups. Cox proportional hazards regression analysis was performed to test the statistical independence and significance between pathological, molecular and clinical variables. Separate analyses were performed using different sets of parameters available either before or after prostatectomy.

## Results

### Technical Issues

A total of 6980 (62.6%) of 11,152 tumor samples were interpretable in our TMA analysis. Reason for non-informative cases (4171 spots; 37.4%) included lack of tissue samples or absence of unequivocal cancer tissue in the TMA spot.

### SFRP4 Expression in Normal and Cancerous Prostate Tissues

**Fig. 1:**
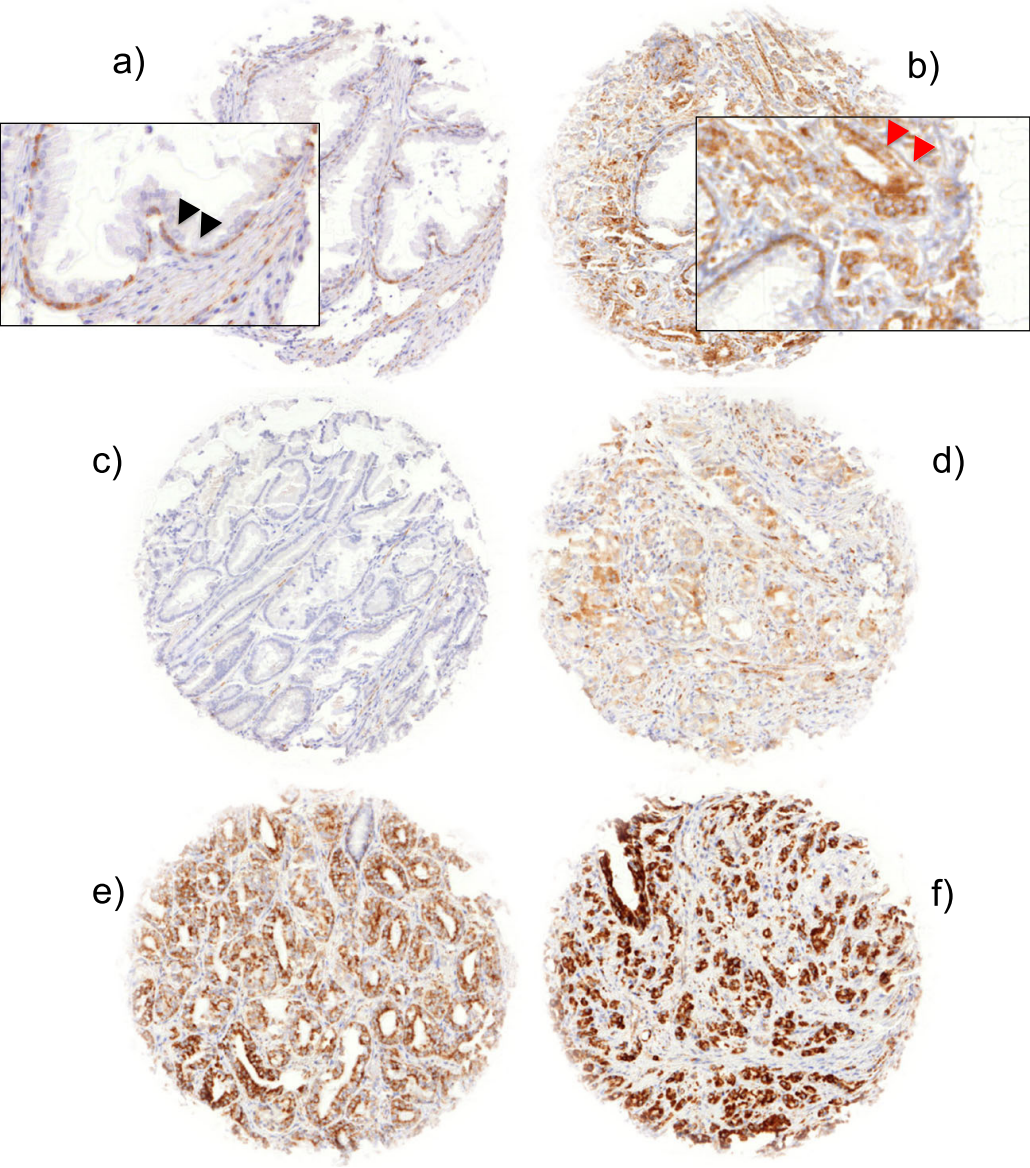
Examples of SFRP4 immunostainings: **a**) SFRP4 basal cell staining in normal prostate glands (black arrowheads) and **b**) cancerous prostate glands (red arrowheads). **c**-**f**) Cancer spots with **c**) lack, **d**) weak, **e**) moderate and **f**) strong staining

**Fig. 2:**
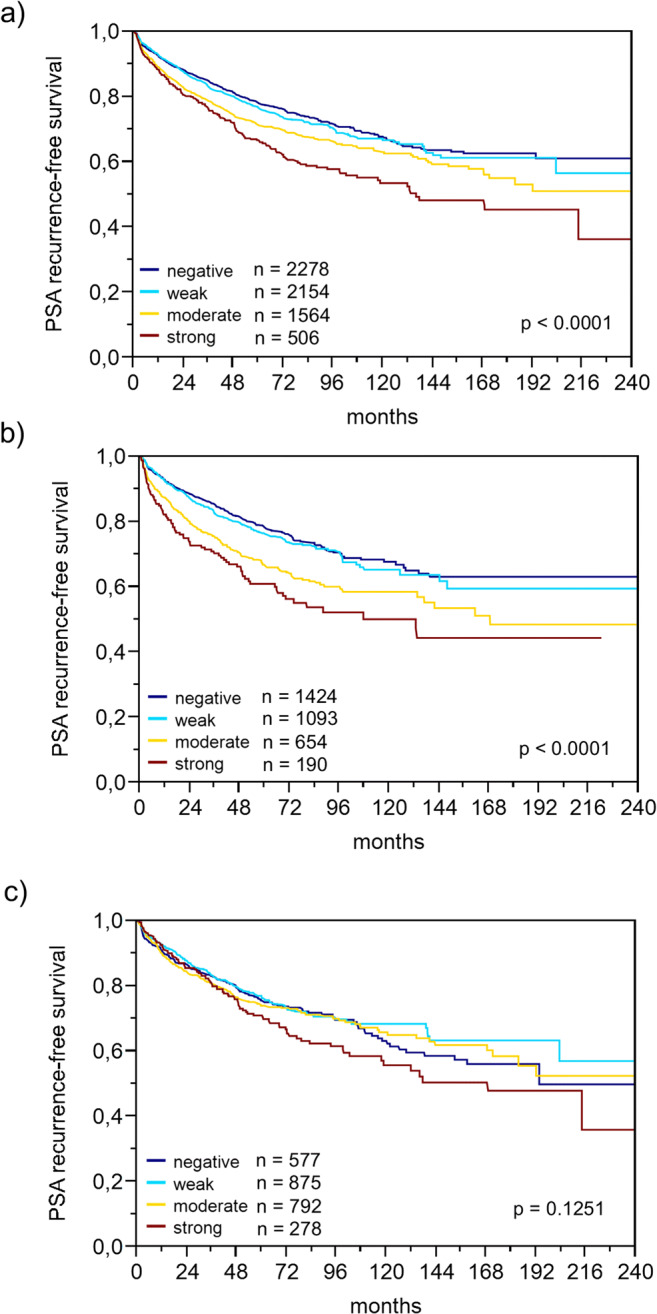
Association between SFRP4 expression and biochemical recurrence in **a**) all cancers, **b**) ERG negative and **c**) ERG positive cancers

SFRP4 showed a granular cytoplasmic immunostaining pattern. Normal prostate gland luminal cells were negative for SFRP4, while basal cells usually showed moderate granular staining. In prostate cancers, SFRP4 staining was seen in 4529 of our 6980 (64.9%) interpretable prostate cancers and was considered weak in 33.2%, moderate in 23.9%, strong in 7.8% of cancers. Representative images of positive and negative SFRP4 immunostainings are given in Fig. 1. Increasing SFRP4 expression was significantly linked to high Gleason grade (*p* < 0.0001), advanced pathological tumor stage (p < 0.0001), positive nodal status (*p* = 0.0002) and positive resection margin status (*p* = 0.0017, Table 2). Strong SFRP4 staining was also strongly linked to early biochemical recurrence (p < 0.0001, Fig. 2a). To exclude a statistical bias because of the high number of samples in our study, we randomly selected three subsets of 2000 cancers each and repeated the analysis. It showed that the prognostic relevance of SFRP4 was retained in these 3 subsets (Supplementary Fig. 1).

**Table 2:** SFRP4 immunostaining and prostate cancer phenotype

Parameter	n Evaluable	Negative (%)	Weak (%)	Moderate (%)	Strong (%)	*p* value
All cancers	6980	35.1	33.2	23.9	7.8	
Tumor stage						
pT2	4422	37.4	34.4	21.7	6.5	<0.0001
pT3a	1652	32.3	32.0	26.3	9.4	
pT3b-pT4	888	29.1	29.1	30.7	11.1	
Gleason grade						
≤3 + 3	1318	42.7	29.7	20.0	7.5	<0.0001
3 + 4	3889	35.1	34.8	23.4	6.6	
3 + 4 Tert.5	302	30.8	38.1	25.5	5.6	
4 + 3	695	31.2	29.8	26.5	12.5	
4 + 3 Tert.5	422	25.1	36.3	29.4	9.2	
≥4 + 4	351	29.9	26.2	31.3	12.5	
Lymph node metastasis						
N0	4284	34.5	32.9	24.2	8.3	0.0002
N+	407	25.6	32.2	31.4	10.8	
Preoperative PSA level (ng/ml)						
<4	748	29.1	36.1	25.3	9.5	0.0008
4–10	4257	34.6	33.7	24.0	7.8	
10–20	1411	38.1	32.1	22.9	6.9	
>20	514	39.9	27.6	24.5	8.0	
Surgical margin						
Negative	5587	35.4	34.0	23.4	7.2	0.0017
Positive	1376	34.1	29.7	25.9	10.2

### SFRP4 and *TMPRSS2:ERG* Fusion Status

**Fig. 3:**
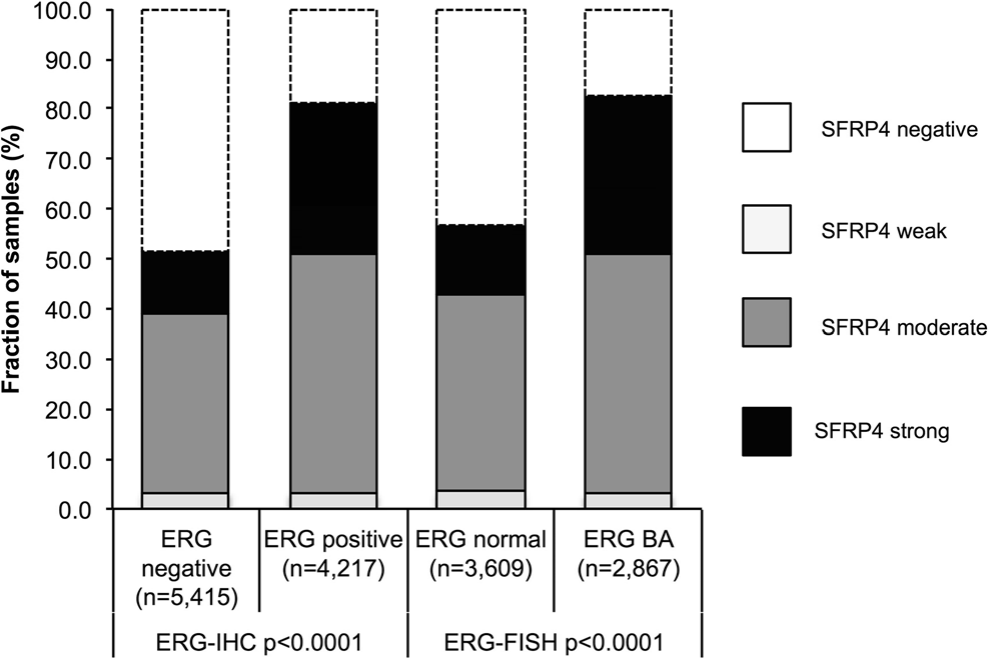
SFRP4 immunostaining and ERG status (IHC/FISH)

Data on *TMPRSS2:ERG* fusion status obtained by FISH were available from 4129 and by immunohistochemistry from 6317 tumors with evaluable SFRP4 immunostaining. Data on both ERG FISH and IHC were concordant in 98.4% of these 4065 cancers with both FISH and IHC data. SFRP4 up-regulation was strongly linked to *TMPRSS2:ERG* rearrangement and ERG expression: Strong SFRP4 positivity increased from 5.6% and 6.3% (by IHC and FISH) in ERG negative cancers to 11.2% and 11.7% in ERG positive cancers (*p* < 0.0001 each, Fig. 3). Because of these differences, associations of SFRP4 with tumor phenotype and PSA recurrence were separately analyzed in ERG negative and ERG positive cancers. It showed that associations between SFRP4 and tumor phenotype (*p* ≤ 0.05 each, Table 3) and PSA recurrence (p < 0.0001, Fig. 2b) were largely driven by the subgroup of cancers lacking ERG fusion. In ERG-positive cancers, an unequivocal association was only found between SFRP4 up regulation and positive surgical margin (*p* = 0.0104, Table 4) but not with any other histological parameter nor with patient prognosis (Fig. 2c).

**Table 3:** SFRP4 immunostaining and prostate cancer phenotype in ERG negative subtype of prostate cancers

Parameter	n Evaluable	Negative (%)	Weak (%)	Moderate (%)	Strong (%)	*p* value
All cancers	3592	42.6	32.5	19.3	5.6	
Tumor stage						
pT2	2360	45.0	33.6	17.0	4.4	<0.0001
pT3a	768	40.9	31.6	20.7	6.8	
pT3b-pT4	458	32.8	28.4	29.0	9.8	
Gleason grade						
≤3 + 3	635	52.8	27.6	14.8	4.9	<0.0001
3 + 4	1927	44.0	34.3	17.7	4.0	
3 + 4 Tert.5	179	35.2	40.2	21.2	3.4	
4 + 3	387	35.4	30.7	22.5	11.4	
4 + 3 Tert.5	241	31.1	34.0	27.4	7.5	
≥4 + 4	221	31.7	26.2	30.8	11.3	
Lymph node metastasis						
N0	2207	41.6	32.5	20.1	5.7	0.0249
N+	209	26.8	29.7	31.6	12.0	
Preop. PSA level (ng/ml)						
<4	323	36.2	35.0	22.0	6.8	0.1201
4–10	2138	42.1	33.6	19.1	5.1	
10–20	809	45.0	30.5	18.8	5.7	
>20	303	46.2	28.1	18.5	7.3	
Surgical margin						
Negative	2878	42.4	33.6	18.8	5.2	0.0111
Positive	709	43.0	28.3	21.3	7.3

**Table 4:** SFRP4 immunostaining and prostate cancer phenotype in ERG positive subtype of prostate cancers

Parameter	n Evaluable	Negative (%)	Weak (%)	Moderate (%)	Strong (%)	*p* value
All cancers	2725	22.6	34.9	31.3	11.2	
Tumor stage						
pT2	1595	23.3	36.6	30.2	10.0	0.0962
pT3a	763	21.4	33.4	32.5	12.7	
pT3b-pT4	359	22.6	30.6	33.7	13.1	
Gleason grade						
≤3 + 3	485	26.6	32.6	28.7	12.2	0.0224
3 + 4	1623	22.2	36.4	31.3	10.1	
3 + 4 Tert.5	102	20.6	35.3	34.3	9.8	
4 + 3	258	21.7	29.1	34.1	15.1	
4 + 3 Tert.5	156	14.7	42.9	30.8	11.5	
≥4 + 4	100	26.0	25.0	35.0	14.0	
Lymph node metastasis						
N0	1692	23.0	34.5	30.5	12.1	0.5362
N+	171	21.6	36.8	32.7	8.8	
Preop. PSA level (ng/ml)						
<4	336	18.8	37.8	31.0	12.5	0.3525
4–10	1721	22.7	34.6	31.1	11.6	
10–20	476	24.4	35.9	30.5	9.2	
>20	171	24.6	29.2	36.3	9.9	
Surgical margin						
Negative	2156	23.4	35.5	30.8	10.3	0.0104
Positive	561	19.8	32.6	33.0	14.6	

### SFRP4 and Genomic Deletions

**Fig. 4:**
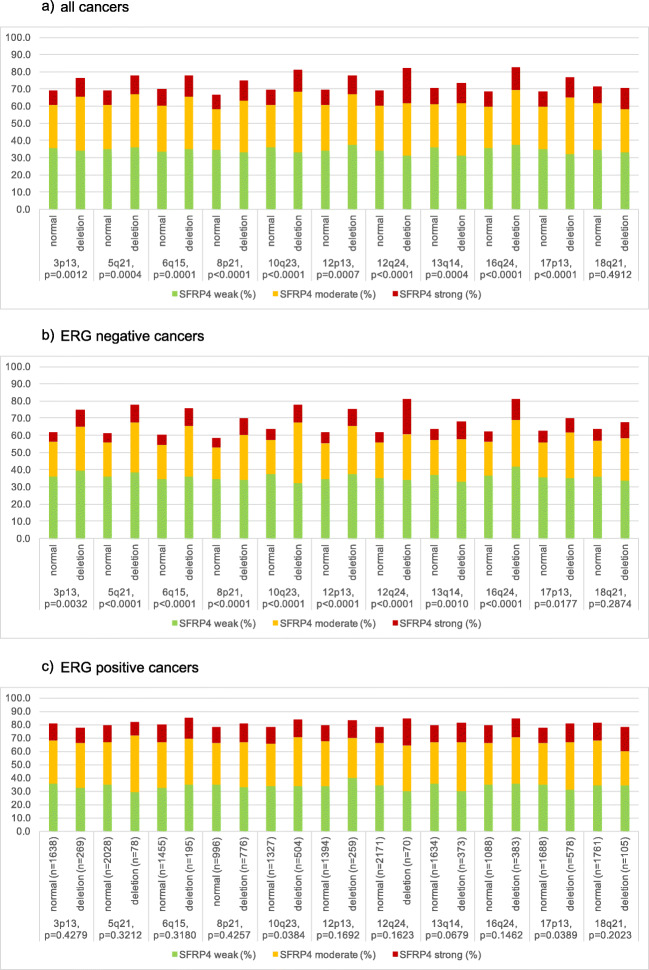
Association between SFRP4 immunostaining and common chromosomal deletions

For 10 of 11 analyzed deletions SFRP4 staining was significantly stronger in deleted than in non-deleted cancers (Fig. 4a). Subset analysis revealed that all these associations were again driven by the subgroup of ERG negative cancers (Fig. 4b) but were largely absent in ERG-positive cancers (Fig. 4c). Among all deletions the relationship between SFRP4 expression and 12q24 deletions stood out. The rate of strongly SFRP4 positive cases was 20.3% in all tumors and 20.3% in ERG negative tumors SFRP4 with 12q24 deletions. These rates dropped to 8.3% in all and 5.9% in ERG negative cancers without 12q24 deletion (*p* < 0.0001 each).

### SFRP4, Androgen Receptor (AR) and Tumor Cell Proliferation (Ki67 Labeling Index)

**Fig. 5:**
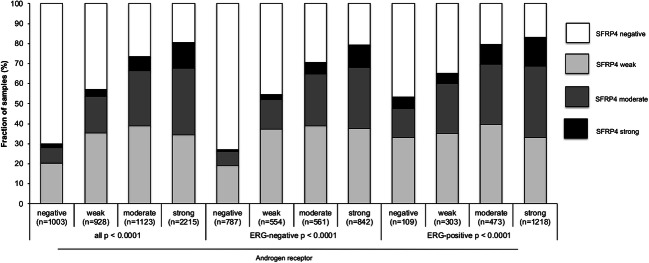
SFRP4 immunostaining and androgen receptor expression

Data on both SFRP4 and AR were available from 5269 cancers. There was a strong positive association between AR expression and SFRP4 staining. Only 7.7% and 1.8% of AR-negative, but 33.1% and 13.1% of strongly AR expressing cancers showed a moderate or strong SFRP4 immunostaining (p < 0.0001). This association held true regardless of the ERG fusion status (p < 0.0001 each; Fig. 5). Strong SFRP4 staining was significantly linked to increased cell proliferation as measured by Ki67 labeling index in all cancers (p < 0.0001). This could also be seen in all tumor subsets of cancers with identical Gleason score (Table 5; *p* ≤ 0.0173).

**Table 5:** SFRP4 immunostaining and Ki67 labeling index

	SFRP4	n=	Ki67LI (mean ± SEM)
all cancers p < 0.0001	negative	1615	1.84 ± 0.07
	weak	1698	2.92 ± 0.06
	moderate	1239	3.45 ± 0.07
	strong	397	3.91 ± 0.13
Gleason ≤3 + 3 *p* < 0.0001	negative	340	1.63 ± 0.11
	weak	252	2.3 ± 0.13
	moderate	186	2.72 ± 0.15
	strong	66	2.29 ± 0.25
Gleason 3 + 4 p < 0.0001	negative	925	1.78 ± 0.07
	weak	1032	2.82 ± 0.07
	moderate	696	3.14 ± 0.09
	strong	206	3.47 ± 0.16
Gleason 3 + 4 Tert.5 *p* = 0.0173	negative	73	2.48 ± 0.28
	weak	80	3.25 ± 0.27
	moderate	66	3.68 ± 0.3
	strong	12	4 ± 0.7
Gleason 4 + 3 p < 0.0001	negative	148	1.91 ± 0.26
	weak	146	3.49 ± 0.26
	moderate	122	3.8 ± 0.29
	strong	59	5.75 ± 0.41
Gleason 4 + 3 Tert.5 *p* = 0.0072	negative	70	2.53 ± 0.42
	weak	120	3.61 ± 0.32
	moderate	86	4.35 ± 0.38
	strong	29	4.52 ± 0.65
Gleason ≥4 + 4 p < 0.0001	negative	59	2.14 ± 0.59
	weak	67	4 ± 0.55
	moderate	82	6.06 ± 0.5
	strong	25	6.68 ± 0.91

### Multivariate Analysis

Multivariate analysis was performed in different scenarios as described before [43] (Table 6): Scenario 1 included postoperatively available parameters (pT, pN, surgical margin status, prostatectomy Gleason grade, preoperative serum PSA, and SFRP4 staining). In scenario 2, pN was omitted because lymph node dissection is not standardized in the surgical therapy of prostate cancer and preferentially done in high-risk patients. Scenario 3 included mainly preoperative parameters (preoperative PSA, clinical tumor stage (cT), prostatectomy Gleason grade, and SFRP4 expression). In scenario 4, the prostatectomy Gleason was replaced by the preoperative biopsy Gleason grade to evaluate the preoperative setting.

**Table 6:** Multivariate Cox regression analysis including established prognostic parameters and the SFRP4 immunostaining in all prostate cancers, in ERG negative and in ERG positive subsets

Tumor subset	Scenario	n analyzable	p -value							
			preoperative PSA-Level	pT Stage	cT Stage	Gleason grade prostatectomy	Gleason grade biopsy	pN Stage	R Stage	SFRP4-Expression
all cancers	1	4361	<0.0001	<0.0001	–	<0.0001	–	<0.0001	<0.0001	0.0027
	2	6462	<0.0001	<0.0001	–	<0.0001	–	–	<0.0001	0.0024
	3	6376	<0.0001	–	<0.0001	<0.0001	–	–	–	<0.0001
	4	6268	<0.0001	–	<0.0001	–	<0.0001	–	–	<0.0001
ERG negative cancers	1	2248	0.0001	<0.0001	–	<0.0001	–	<0.0001	0.2283	0.0086
	2	3336	<0.0001	<0.0001	–	<0.0001	–	–	0.02	0.0014
	3	3305	<0.0001	–	<0.0001	<0.0001	–	–	–	0.0001
	4	3253	<0.0001	–	<0.0001	–	<0.0001	–	–	<0.0001
ERG-positive cancers	1	1730	0.0094	<0.0001	–	<0.0001	–	0.0856	0.0003	0.4645
	2	2513	0.0001	<0.0001	–	<0.0001	–	–	<0.0001	0.7440
	3	2465	<0.0001	–	<0.0001	<0.0001	–	–	–	0.1938
	4	2421	<0.0001	–	<0.0001	–	<0.0001	–	–	0.035

**Fig. 6:**
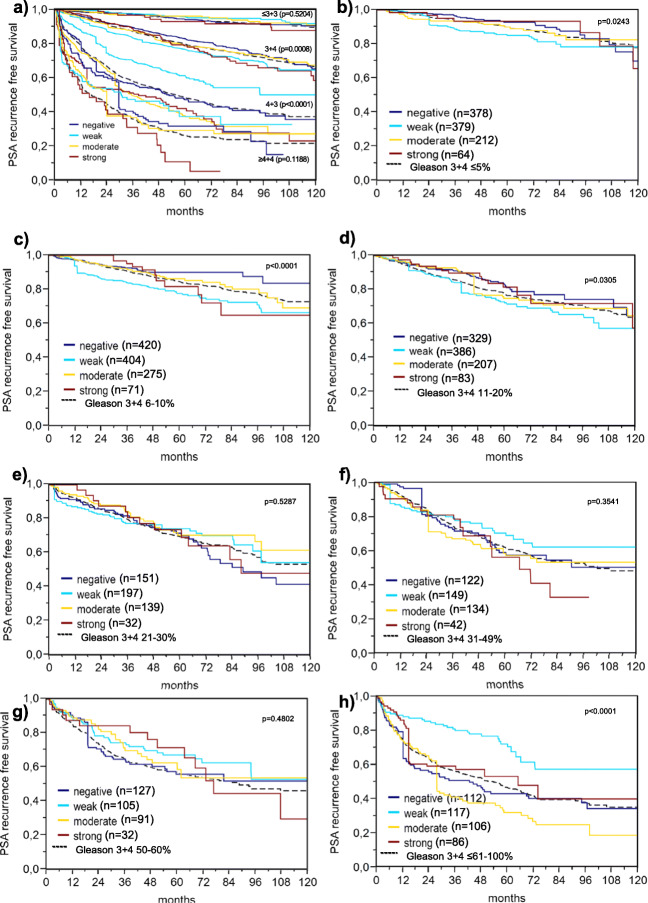
Prognostic impact of SFRP4 defined by the Gleason score. **a** Impact of SFRP4 expression as compared to the classical Gleason score categories. **b**-**h** Impact of expression as compared to the quantitative Gleason score categories defined by subsets of cancers with **b**) ≤5% Gleason 4 patterns, **c** 6–10% Gleason 4 patterns, **d** 11–20% Gleason 4 patterns, e) 21–30% Gleason 4 patterns, f) 31–49% Gleason 4 patterns, g) 50–60% Gleason 4 patterns, h) ≥61% Gleason 4 patterns

SFRP4 measurement provided independent prognostic information in all scenarios when all cancers were jointly analyzed (*p* ≤ 0.0027) as well as in the subset of ERG-negative cancers (*p* ≤ 0.0086, Table 6). Subset analysis of cancers with identical Gleason score revealed a prognostic role of SFRP4 in subsets of cancers with Gleason grade 4 + 3 (*p* = 0.0005, Fig.6a). However, SFRP4 expression lacked unequivocal prognostic impact in tumor subsets defined by a quantitative Gleason grade (Fig. 6b-h). The hazard ratios and 95% confidence intervals are shown in the Supplementary Table 1.

## Discussion

The results of our analysis demonstrate that SFRP4 up-regulation is an independent predictor of early PSA recurrence in prostate cancers lacking *TMPRSS2:ERG* fusions.

The granular cytoplasmic staining observed in our study fits well to the assumed localization of the secreted SFRP4 in exocytic membrane vesicles. Higher levels of SFRP4 staining in cancer glands as compared to adjacent normal prostate gland demonstrate that SFRP4 upregulations parallels prostate cancer development and progression. This is also supported by a recent meta-analysis including mRNA expression data from 8 studies downloaded from Gene Expression Omnibus (GEO) and The Cancer Genome Atlas (TCGA), as well as immunohistochemistry results from 40 patients [19]. In the latter work, Sandsmark et al. reported higher immunohistochemistry staining levels of SFRP4 in prostate cancer samples as compared to normal samples, and also found that four out of five mRNA expression studies, which included both normal and cancer tissues, observed SFRP4 up regulation in the tumor samples [19].

SFRP4 upregulation was clearly linked to adverse tumor features in our study, including advanced stage, high Gleason grade, nodal metastases, rapid tumor progression (as measured by the Ki67 labeling index) and early biochemical recurrence. Similar findings were reported by Sandsmark et al. and also in five of six cohorts with SFRP4 mRNA data and biochemical recurrence as endpoints [19]. Another mRNA expression study described SFRP4 as one of the most deregulated genes out of 40 Wnt-pathway related genes analyzed in 54 prostate cancers [44]. Only one study found a link between high SFRP4 expression and favorable prognosis. Using a homemade sheep polyclonal antibody, Horvath et al. found that accumulation of SFRP4 immunostaining at the cell membrane was linked to a prolonged recurrence-free interval in a cohort of 229 patients treated for radical prostatectomy [16]. We did not observe any SFRP4 membrane staining in our study using a commercial rabbit monoclonal antibody. Given the known functions of the protein we would not expect membranous staining in case of SFRP4. SFRP4 is typically described as a tumor suppressor gene with a role in many cancer types. This is based on its inhibitory effect on Wnt signaling [14, 15]. Functional studies in prostate cancer cell lines further showed that SFRP4 overexpression resulted in a reduced cellular proliferation, anchorage-independent growth, and invasiveness [16, 17]. Finding strong associations between the putative tumor suppressor gene SFRP4 up regulation and aggressive tumor behavior in clinical cancer specimens is thus not intuitive. Compensatory SFRP4 upregulation in case of a highly activated Wnt pathway represents a likely explanation for high SFRP4 levels in aggressive cancers. It is possible, that SFRP4 is upregulated in such cancers in an attempt to regain growth control. P16 overexpression in human papilloma virus induced cervical carcinoma represents a well-studied example of a compensatory overexpression of a tumor suppressor gene [45]. The notion, that SFRP4 upregulation may indicate altered Wnt signaling could also explain the strong link of high SFRP4 expression with high tumor cell proliferation and 10 of 11 analyzed chromosomal deletions. Several studies have demonstrated, that inappropriate activation of Wnt signaling can induce chromosomal instability with formation of translocations and chromosomal deletions [46–49].

Our analysis of molecularly defined tumor subgroups revealed that the prognostic impact of SFRP4 expression was almost entirely driven by the ERG negative cancer subgroup. There may be several non-exclusive explanations for this finding. First, compensatory SFRP4 upregulation might also appear in aggressive cancers with activated Wnt signaling due to reasons other than ERG fusion. Second, the tumor biological relevant role of SFRP4 may be abrogated after ERG activation, for example, if ERG target genes become expressed that interfere with SFRP4 function. Third, given that at least some other SFRP family members can have both suppressive or activating roles on Wnt signaling activity depending on their individual expression levels [50], it cannot be excluded that upregulated SFRP4 might have a Wnt activator role specifically in ERG-negative cancers. However, functional analysis are required to validate these scenarios.

*TMPRSS2:ERG* fusions are observed in about 50% of prostate cancer [51, 52] resulting in permanent overexpression of the transcription factor ERG [38]. ERG overexpression by itself is not prognostic in surgically treated patients [30]. Since ERG modulates the expression of more than 1600 genes, it is difficult to predict its biological effect. The strikingly higher SFRP4 expression in ERG positive (>30% with strong SFRP4 positivity) than in ERG negative cancers (<15% with strong SFRP4 positivity) could be explained by the transcriptional activation of ERG and its direct target EZH2 [53], known reported as the upstream regulator of SFRP4 [54]. However, it cannot be excluded that the SFRP4 up regulation reflects a compensatory cellular mechanism. Several studies report ERG activation as a Wnt signaling inducer [55, 56] through frizzled-4 (FZD4) player, a direct antagonist of the Wnt inhibitory protein SFRP4 [57]. The striking correlation of SFRP4 and AR expression fits well with the established functional interaction of androgen receptor signaling and the Wnt pathway [58].

Whether a different prognostic outcome between the ERG- positive and negative subset of a biomarker is due to direct biological effects of ERG on the biomarker or indirect by blurring the expression scale due to ERG-induced higher expression of the biomarker is difficult to discern. n favor for the latter argument we did not observe an inverse correlation between the ERG- positive and negative subset for any of the about 100 different biomarkers tested so far. SFRP4 belongs to the few biomarkers, which were found to be prognostic in either ERG positive [59, 60] or ERG negative [61–63] cancers but not in both groups.

SFRP4 is part of a commercially available RNA based prognostic gene expression panel. The impact of ERG expression on the prognostic impact of SFRP4 expression raises the question whether this commercial prognosticator may also be dependent on the ERG status. The physiologic expression of SFRP4 in basal cells of non-neoplastic glands constitutes another problem for using SFRP4 RNA expression as a prognostic test. The majority of clinical prostate cancer samples contain a variable fraction of non-neoplastic glands which will lead to a variable impact on the SFRP4 RNA measurement. The data of this study suggest, however that SFRP4 protein measurement may result in clinically useful prognostic information in ERG negative cancer. In this subgroup, SFRP4 expression had a significant prognostic impact which was independent of established prognostic parameters, irrespective of whether all available features or only preoperatively available prognostic parameters were included into the analysis. That SFRP4 expression still largely lacks prognostic impact in cancers with identical traditional quantitative Gleason grade demonstrates the statistical power of a thorough morphological assessment. While SFRP4 expression measurement alone might not be sufficiently prognostic to support clinical decision making, it is of note that studies by us and others have recently identified a good number of prognostic protein markers, such as for example AZGP1 [64], EZH2 [65], p62 [66], GSK3ß [67], PSCA [68], and PTEN [36]. Especially in the light of recent technical developments enabling a simultaneous immunohistochemical analysis of up to 6 antibodies [69], we expect, that immunohistochemical expression panels may be developed for prostate cancer prognosis assessment. SFRP4 may well become an element of such a test for ERG negative prostate cancer.

In summary, upregulation of SFRP4 is associated with adverse tumor features, genomic instability and poor patient prognosis in ERG negative prostate cancer. SFRP4 expression analysis may have prognostic utility either alone, or more likely, in combination with other biomarkers.

## Electronic supplementary material

Suppl. Table 1 The hazard ratios and 95% confidence intervals and SFRP4 expression (XLSX 9 kb)

Suppl. Fig. 1 Correlation between SFRP4 expression and biochemical recurrence in three different validation subsets of 2000 randomly analyzed cancers (subset 1: a), subset 2: b), subset 3: c)) (PDF 155 kb)

## Abbreviations

AR: Androgen receptor
BA: Break-Apart
CDKN1B: Cyclin Dependent Kinase Inhibitor 1B
CHD1: Chromodomain-Helicase-DNA-Binding Protein 1
ERG: ETS Transcription Factor ERG
FISH: Fluorescence in-situ hybridization
FOXO1: Forkhead Box Protein O1
FOXP1: Forkhead Box Protein P1
IHC: Immunohistochemistry
Ki67LI: Ki67 labeling index
MAP3K7: Mitogen-Activated Protein Kinase Kinase Kinase 7
PSA: Prostate specific antigen
PTEN: Phosphatase and tensin homolog
RB1: RB Transcriptional Corepressor 1
TMPRSS2: ERG: Transmembrane protease, serine 2: ETS-related gene fusion
TMA: Tissue microarray
TP53: Tumor protein P53
