# Supplementary figures and images for: Secreted Frizzled-Related Protein 4 (SFRP4) Is an Independent Prognostic Marker in Prostate Cancers Lacking TMPRSS2: ERG Fusions
Source: Pathol Oncol Res. 2020 Jul 16;26(4):2709–22. doi: 10.1007/s12253-020-00861-9 (PMC7471174; doi:10.1007/s12253-020-00861-9)

Suppl. Fig. 1

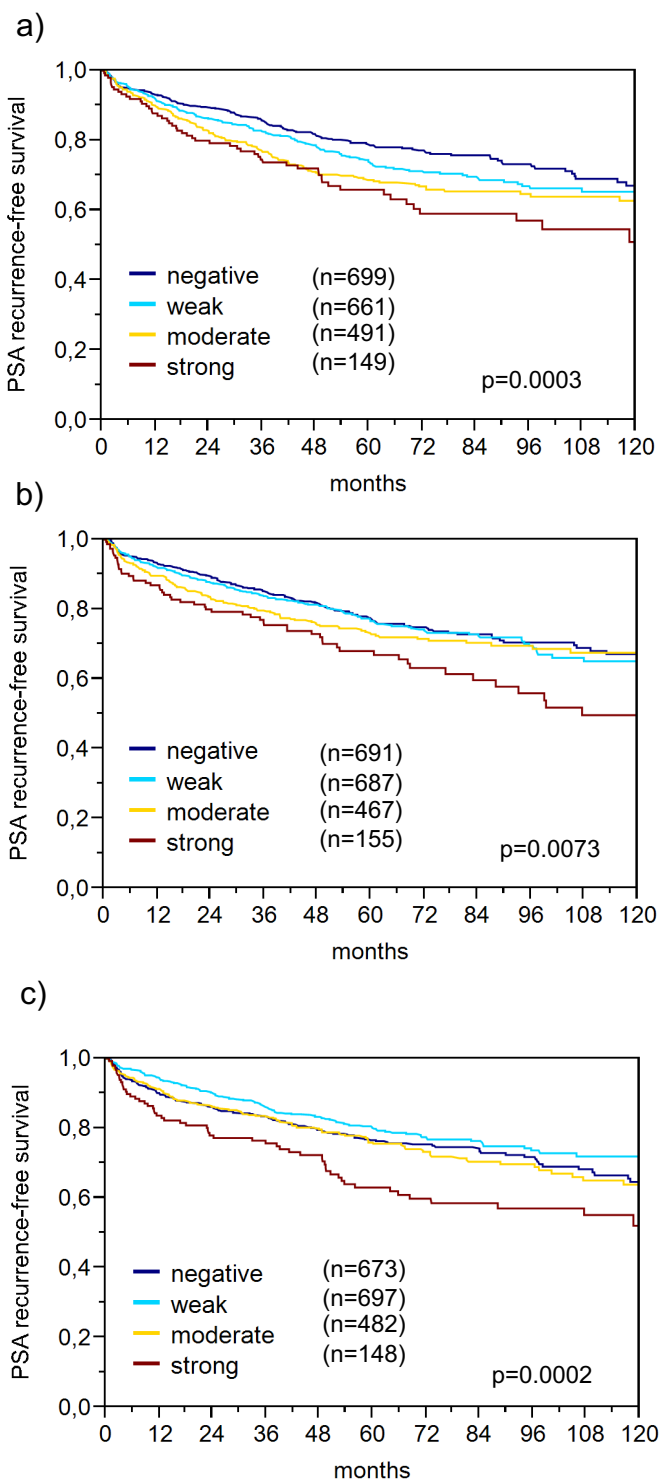

Supplement: Supplementary file 2 — Correlation between SFRP4 expression and biochemical recurrence in three different validation subsets of 2000 randomly analyzed cancers (subset 1: a), subset 2: b), subset 3: c)) (PDF 155 kb) [file 12253_2020_861_MOESM2_ESM.pdf]
